# Interaction Effects of *FTO* and *MC4R* Polymorphisms on Total Body Weight Loss, Post-Surgery Weight, and Post-Body Mass Index after Bariatric Surgery

**DOI:** 10.3390/genes15040391

**Published:** 2024-03-22

**Authors:** Elva Perez-Luque, Edgar S. Daza-Hernandez, Nicte Figueroa-Vega, Monica I. Cardona-Alvarado, Norberto Muñoz-Montes, Claudia Martinez-Cordero

**Affiliations:** 1 Departamento de Ciencias Médicas, Campus León, Universidad de Guanajuato, León 37320, Guanajuato, Mexico; 2 Hospital Regional de Alta Especialidad del Bajío, León 37660, Guanajuato, Mexico

**Keywords:** obesity, bariatric surgery, interaction of *FTO* genotypes, TBWL, post-surgery

## Abstract

Bariatric surgery (BS) is considered the most effective intervention for patients with severe obesity and is used to maintain long-term weight loss and glycemic control. The aim of this study was to analyze the effects of genotypes and haplotypes of the fat mass and obesity-associated (*FTO*) and melanocortin 4 receptor (*MC4R)* genes on total body weight loss (TBWL), post-surgery weight, and post-BMI after bariatric surgery. We retrospectively selected 101 patients from Bajio High Specialty Regional Hospital, León Guanajuato, México, who underwent Roux-en-Y gastric bypass (RYGB) to determine their body mass index (BMI), blood pressure, biochemical characteristics, and comorbidities. Post-surgery, patients were referred for registered anthropometry and blood pressure. Glucose, lipid and hepatic profiles, and insulin, leptin, and ghrelin levels were measured, and rs9939609, rs9930506, and rs1421085 *FTO* and rs17782313 *MC4R* polymorphisms were genotyped. Six (4–8) years after BS, post-surgery weight was greater in carriers of the rs9939609 and rs1421085 risk genotypes. TBWL was lower for the rs9930506 and rs1421085 risk genotypes. Insulin and HOMA-IR were greater in patients with the three *FTO* polymorphisms. There were significant interaction effects of the rs9930506 and rs1421085 *FTO* risk genotypes on weight and BMI in response to BS. No association was found with the *MC4R* polymorphism. The genotypes and haplotypes of the *FTO* gene influence post-surgery weight, TBWL, insulin levels, and HOMA-IR.

## 1. Introduction

Obesity is associated with increased morbidity and mortality and is a risk factor for cardiovascular disease. Therefore, preventing and treating obesity has become a major public health goal [1]. Bariatric surgery is considered the most effective intervention for patients with severe obesity (BMI ≥ 40 kg/m^2^ or ≥35 kg/m^2^) with comorbidities [2] and for maintaining weight loss and glycemic control in the long term [3]. In addition, weight loss, improvement, or long-term remission of comorbidities such as T2D, hypertension, and dyslipidemia after bariatric surgery has been reported [4].

One of the most effective surgical methods for treating severe obesity is Roux-en-Y gastric bypass (RYGB), which has been well documented to achieve sustained long-term results [2]. Nevertheless, some studies have reported that 20–30% of patients do not achieve satisfactory weight loss after bariatric surgery [3,5]. Furthermore, another study reported that one-fifth of patients undergoing bariatric surgery may not lose enough weight to be considered successful [6]. Several factors associated with minor weight loss have been described, such as behavioral problems, social and demographic factors, surgical technique, and genetic polymorphisms [7]. Weight loss in 100 patients 13–15 years after bariatric surgery has been reported (mean 29.5 kg ranging from −13.6 to 93.6 kg), demonstrating the marked variability in surgery-induced long-term weight loss [8].

The first genome-wide association study of weight loss in response to RYGB at 2 years after surgery showed that variation in weight loss outcomes after RYGB may be influenced by several common genetic variants in genes with potential biological relevance, including *PKHD1*, *HTR1A*, *NMBR*, and *IGF1R* [9]. However, the possibility that genetic factors affect the success of bariatric surgery remains to be investigated. It is unclear whether DNA sequence variation in genes related to obesity, such as the *FTO* and *MC4R* genes, affects the outcome of weight loss intervention. Given the known associations between the *FTO* gene and obesity, it is important to examine the role of variants in this gene in bariatric surgery outcomes. A previous study, involving 146 patients who underwent Roux-en-Y gastric bypass and were genotyped for rs9939609 to assess its role in postoperative weight gain, revealed that 71.2% of patients carried at least one risk allele. Patients with one or two risk alleles (TA or AA) had increased body weight and BMI at 3, 4, and 5 years following surgery compared with TT patients [10]. At 36, 48, and 60 months after bariatric surgery, body weight, fat mass, and body mass index (BMI) were greater, while excess body mass loss (EBWL) was lower in carriers of the rs9939609 risk allele [11]. A study performed on 168 Mexican mestizos and 81 patients with other ancestral origins showed a statistically significant association of rs9939609 with smaller changes in postoperative %EBWL and BMI [12]. The results of a study in the Italian population demonstrated a strong association between *FTO* rs9939609 (*p* < 0.043) and rs9930506 (*p* < 0.029) and BMI. *FTO* rs9930506 was significantly associated with increased BMI in a G allele dose-dependent manner (BMI + 1.4 kg/m^2^ per G allele) [13].

An association of the rs1421085 polymorphism between at-risk alleles and high-fat refined starch intake was observed in 133 overweight and obese Caucasian individuals [14]. In addition, obesity risk alleles at *FTO* rs1421085 significantly predicted more daily eating episodes (*p* = 0.001) [15]. The effect of polymorphisms rs1421085 and rs9930506 of FTO on the success of bariatric surgery has rarely been studied. Melanocortin-4 receptor (*MC4R*) mutations are the most frequent monogenic causes of severe early onset human obesity [16]. rs17782313 is associated with obesity; however, in a case–control study, this polymorphism did not affect weight loss or body composition over one year after bariatric surgery [17]. However, women with severe obesity carrying this polymorphism presented a higher pre-surgical BMI; therefore, they are more unlikely to have a nonobese BMI (<30 kg/m^2^) [18].

Therefore, identifying genetic factors related to weight loss during bariatric surgery may help to guide weight management strategies before and after surgery. The objective of this study was to analyze the effect of rs9939609, rs1421085, and rs9930506 of the *FTO* gene and rs17782313 of the *MC4R* polymorphism on changes in weight, BMI, TBWL, and metabolic variables after bariatric surgery.

## 2. Materials and Methods

### 2.1. Participants

We performed a retrospective analysis of the information contained in the medical electronic files of patients from the surgery service of the Bajio High Specialty Regional Hospital, León Guanajuato, México, who underwent Roux-en-Y gastric bypass (RYGB) from May 2010 to November 2021, resulting in 169 patients being registered. Of them, 101 agreed to participate in this study, including 78 women and 23 men. From the medical files of the patients, we collected personal and clinical data, and the conditions before bariatric surgery, such as weight, body mass index (BMI), systolic blood pressure (SBP), diastolic blood pressure (DBP), fasting glucose, lipid profiles, comorbidities, hypertension, dyslipidemia, and diabetes, were recorded. We fully informed all participants of the aims of this study, and we asked them for signed informed consent to participate in this study. This study was carried out according to the ethical standards of the Declaration of Helsinki (1983) and in agreement with the Good Clinical Practice guidelines. This study was approved by the Investigation Committee of Bajio High Specialty Regional Hospital (CI/HRAEB/044/2020-CEI/HRAEB/36/2020) and by the Institutional Ethics Committee of the University of Guanajuato (CIBIUG-P70-2020), Guanajuato, México.

### 2.2. Post-Surgery Procedure

The patients were assessed at 8 AM after an overnight fast. Personal and clinical data were registered, anthropometric measurements were taken, and blood pressure (BP) was measured. Weight was measured with a Roman-type Tanita BC533 scale, and height was measured using a SECA 406 Stadiometer to calculate BMI (kg/m^2^). Changes in BMI (%BMI), total body weight loss (TBWL), percent of total body weight loss (%TBWL), and percent of excess body weight loss (%EBWL), in which ideal weight is defined by the weight corresponding to a BMI of 25 kg/m^2^, were calculated according to Brethauer et al. [19]. Systolic and diastolic blood pressures were measured in a sitting position after ten minutes of rest. All measurements were conducted in duplicate. Venous blood samples were taken after overnight fasting for the measurement of serum glucose and lipid profiles, hepatic proliferation, and serum insulin, leptin, and ghrelin levels and for DNA extraction. Serum glucose and lipid and hepatic profiles were measured using enzymatic methods with a semiautomatic chemical analyzer (SPINLAB SPINREACT). Insulin, leptin, and ghrelin concentrations were measured using ELISA kits (ALPCO Immunodiagnostic AG, Stubenwald-Allee, Bensheim, Germany) with a sensitivity of 0.399 µIU/mL (range of 3.0–200 µIU/mL) and 3.2 and 5.7% CV for insulin, a sensitivity of 0.42 ng/mL (normal range of 1–100 ng/mL) and 3.5 and 5.9% CV for leptin, and a sensitivity of 1.0 pg/mL (0–5000 pg/mL) and 8% CV for ghrelin. Insulin resistance was calculated using the homeostasis model assessment (HOMA-IR) with the formula fasting glucose × fasting insulin/22.5 (U/L) [20].

### 2.3. Single Nucleotide Polymorphism Genotyping

Genomic DNA was isolated from peripheral blood leucocytes according to the TSNT protocol and quantified using a NanoDrop system (Roche). Genotyping of rs9939609, rs9930506, and rs1421085 *FTO* and rs17782313 *MC4R* polymorphisms (SNPs) was carried out with quantitative polymerase chain reaction (qPCR) using validated TaqMan^®^ probes and TaqMan™ Genotyping Master Mix enzyme (Thermo Fisher Scientific, Inc., Waltham, MA, USA) following the supplier’s specifications.

For rs9939609 (VIC/FAM), the A/T transversion substitution was GGTTCCTTGCGACTGCTGTGAATTT[A/T]GTGATGCACTTGGATAGTCTCTGTT; for rs9930506 (VIC/FAM), the A/G transition substitution was AGGGACACAAAAAGGGACATACTAC[A/G]TGAATTACTAATATCTAAGAAAATA; for rs1421085 (VIC/FAM), the C/T transition substitution was TAGCAGTTCAGGTCCTAAGGCATGA[C/T]ATTGATTAAGTGTCTGATGAGAATT; and for rs17782313 (VIC/FAM), the C/T transition substitution was GTTTAAAGCAGGAGAGATTGTATCC[C/T]GATGGAAATGACAAGAAAAGCTTA. In addition, allelic discrimination and data were analyzed by the CFX96 Touch Real-Time PCR Detection System (Bio-Rad Laboratories, Inc., Hercules, CA, USA).

### 2.4. Statistical Analysis

We assessed the normality of the distribution of the data by the Kolmogorov–Smirnov test and evaluated the data for Hardy–Weinberg equilibrium. We present the data as the mean ± standard deviation (SD) or median (25–72 quartiles) using descriptive statistics. We compared groups using the *t* test, Mann–Whitney test, or ANOVA according to the distribution of the data. To compare categorical variables, we used chi-squared or Fisher’s exact tests. To evaluate the interaction effects of *FTO* polymorphisms with weight and BMI, we performed a variance analysis of repeated measures of one factor. A *p* value of <0.05 was considered significant. Analyses were carried out using the statistical Statistica 7 package (StatSoft Inc., Tulsa, OK, USA).

## 3. Results

We included a total of 101 patients—78 women and 23 men—from July to December 2021 at the Surgery Service of Bajio High Specialty Regional Hospital (HRAEB), Leon, Guanajuato, Mexico, with a median of 6 (4–8) years after bariatric surgery. Table 1 shows the anthropometric, clinical, and metabolic characteristics of the post-surgery patients. In this cohort, the TBWL was 34.7 (22.9–48.9), the %TBWL was 23 (16.6–33), the %EBWL was 58.5 (40–79), and the “ideal” weight was 64 (58.5–68.9).

Table 2 shows the genotypic and allelic frequencies of the four polymorphisms in the cohort studied. All polymorphisms were analyzed according to Hardy–Weinberg equilibrium.

To evaluate the effect of *FTO* polymorphisms on anthropometric and metabolic variables, we compared the genotypes according to the dominant model 6 (4–8) years after bariatric surgery. Table 3 shows that all three polymorphisms were significantly different.

*rs9939609* (T>A) *FTO polymorphism*.

Six years after BS, the carriers of one or two risk alleles of the rs9939609 (TT vs. TA and AA) polymorphism had 8.6 kg more and higher serum insulin, HOMA-IR, and serum leptin levels than did the wild type.

*rs9930506* (A>G) *FTO polymorphism*.

In the carriers of one or two risk alleles of the rs9930506 polymorphism under the dominant model (AA vs. AG and GG), we observed that pre-surgery BMI and TBWL were lower in carriers of the AG and GG genotypes than in AA carriers. However, the total cholesterol, insulin, and insulin resistance levels were greater than those in the wild type (Table 3).

*rs1421085* (T>C) *FTO polymorphism*.

According to the dominant model, carriers of one or two risk alleles, *rs1421085* (TC or CC), had significantly greater post-surgical weight, serum insulin, and insulin resistance than did carriers of the TT genotype. The carriers of the TC and CC genotypes also lost less total weight (TBWL) than did those of the TT genotype (Table 3).

*rs17782313* (T>A) *MC4R polymorphism.*

No significant differences were found in the comparison among the analyzed variables with different genotypes of the rs17782313 MC4R polymorphism under dominant and codominant models.

*Analysis of FTO haplotypes.*

*This analysis revealed haplotypes with two FTO polymorphisms.*

*Analysis of the rs9939609 (T>A) and rs1421085 (T>C) haplotypes.*

Among the carriers of the rs9939609A and rs1421085C risk haplotypes of *FTO* polymorphisms, the TBWL and %EBWL were significantly lower than those among the other haplotypes (F = 3.42, *p* = 0.020 and F = 16.73.47, *p* = 0.018, respectively).

No significant differences in the comparison among anthropometric, metabolic, and hormonal variables with the rs9930506 (A>G) and rs1421085 (T>C) haplotypes of these polymorphisms were found.

*Analysis of the rs9939609 (T>A) and rs9930506 (A>G) haplotypes.*

The carriers of the rs9939609A and rs9930506G risk haplotypes lost less total weight (TBWL). The %EBWL was greater for haplotype 3. Serum insulin and HOMA-IR were significantly greater in haplotype 4 than in haplotypes 1 and 3. Haplotype 2 did not show significant differences from the other haplotypes (Table 4).

Interaction effects of FTO polymorphisms on weight and BMI in bariatric surgery patients.

Significant interaction effects of the rs9930506 and rs1421085 FTO risk genotypes on weight and BMI in response to bariatric surgery were found. No interaction effects of bariatric surgery on the rs9939609 risk genotype were found (Table 5).

## 4. Discussion

In this work, we analyzed the effects of the rs9939609, rs1421085, and rs9930506 risk genotypes of the *FTO* gene and the rs17782313 *MC4R* gene, as well as the risk haplotypes of the *FTO* gene on pre-surgery weight, post-surgery weight, pre-BMI, post-BMI, TBWL, %EBWL, and metabolic conditions after a median of 6 (4–8) years after bariatric surgery. Successful bariatric surgery in terms of weight outcomes has been described as >50% excess weight loss (%EWL), 20–30% loss of initial weight, or a BMI < 35 kg/m^2^ [21]. Our cohort maintained a %TBWL of 58.5 (40–79) and a BMI of 33.8 (29–38.8) 6 years after bariatric surgery. In addition, all post-surgery metabolic parameters corresponded to individuals without metabolic problems and important remission of T2DM and hypertension. Nevertheless, the patients had moderately satisfactory results in weight loss, and %EBWL and %TBWL did not reach their ideal weight. Our data still show clinical variability in patient outcomes after RYGB.

In patients with severe obesity who underwent laparoscopic mini-gastric bypass (LMGB), the rs9939609 genotype was associated with a significant decrease in BMI and a significant improvement in HbA1c levels [22]. An association of the risk rs9939609 genotype with smaller changes in postoperative BMI and %EBWL in patients with obesity after RYGB has been reported [12]. The results of another study suggested that the *FTO* gene may play an important role in the long-term outcomes of bariatric surgery given that body weight, fat mass, and BMI are greater while EBWL is lower in carriers of the rs9939609 *FTO* risk allele [11]. In our work, post-surgery weight and insulin, leptin, and insulin resistance levels were significantly greater in carriers of one or two risk alleles, but we did not find differences in EBWL.

The effect of the rs9930506 FTO polymorphism on outcomes after bariatric surgery has been briefly explored. In our previous work involving 15 patients with severe obesity, before bariatric surgery, we found that carriers of one or two risk alleles of the polymorphism weighed 34 kg more than did wild-type carriers. After sleeve gastrectomy (SG), carriers of one or two risk alleles lost more weight (37 kg) than did wild-type carriers (13.7 kg), and similar results were observed for BMIs of 11.5 vs. 5.2 kg/m^2^ at 6 months of SG [23]. In this work, the pre-surgery BMI was low in carriers of one or two risk alleles; however, they lost less TBWL. Total cholesterol, insulin, and insulin resistance were greater in carriers of one or two risk alleles than in wild-type carriers. These data suggest a possible effect of rs9930506 on the outcomes of bariatric surgery [23].

Several studies have confirmed the association of rs1421085 FTO with the risk of developing obesity [24]. We found that carriers of the risk TC and CC genotypes had greater post-surgical weight and lower TBWL than carriers of the TT genotype. These results could be explained in part by a previous study at the molecular level, which reported that the rs1421085 variant alters the conserved motif for the ARID5B repressor, which leads to depression of a potent preadipocyte enhancer and a doubling of IRX3 and IRX5 expression during early adipocyte differentiation, resulting in a shift from beige adipocytes to white adipocytes and a reduction in mitochondrial thermogenesis and an increase in lipid stores. Repair of the ARID5B motif and editing of rs1421085 in primary adipocytes from a patient with the risk allele restored IRX3 and IRX5 expression and activated the browning expression program [25]. We found that insulin and HOMA-IR levels were greater in individuals with the TC and CC risk genotypes than in those with the wild-type genotype as a consequence of greater post-surgery weight and lower TBWL in these genotypes.

Interestingly, we observed a common result in three polymorphisms of the FTO gene and its relationship with higher insulin and HOMA-IR levels in individuals with different risk genotypes. In this work, rs17782313 of the *MC4R* gene was not associated with BMI or TBWL or with changes in any variables studied after bariatric surgery. According to our results, another study showed that *MC4R* mutations and polymorphisms do not affect weight loss or body composition over 1 year after BS [26]. However, this polymorphism has been associated with greater intake of total energy and dietary fat, greater long-term weight changes, and increased risk of diabetes in women [27].

Our data revealed a significant effect of the rs9939609A and rs1421085C risk haplotypes of the *FTO* gene, which are associated with low TBWL and %EBWL. The rs9939609A and rs9930506G risk haplotypes also showed an effect on TBWL that manifested with less loss of TBWL and with higher insulin and insulin resistance levels after 6 (4–8) years. Interestingly, the variance analysis of repeated measures of one factor showed interaction effects of the rs9930506 and rs1421085 *FTO* polymorphisms on weight and BMI after bariatric surgery. This is the first report of the interaction effects of these *FTO* polymorphisms with anthropometric changes in BS patients. In the literature, we found only one study by Harbron J et al. that showed an association of the *FTO* rs1421085 and 17817449 haplotypes with dietary intake, eating behavior, and psychological health [14].

In our work, insulin and HOMA-IR were greater in the risk genotype group than in the wild-type group for three polymorphisms (rs9939609, rs9930506, and rs1421085) of the *FTO* gene after BS. In a previous report, the rs9939609A risk allele was significantly associated with increased plasma insulin levels (*p* = 0.05) and increased homeostasis model assessment (HOMA-IR) (*p* = 0.02) [28]. Another report showed that the rs9939609 risk allele was associated with increased serum leptin and decreased HDL levels in overweight people [29]. The presence of the T allele in the two rs9939609 and rs17817449 polymorphisms in the *FTO* gene was associated with an increased risk for the development of T2D in Iraqi individuals with obesity. In relation to the phenotypic parameters, these two polymorphisms were significantly associated with increased BMI, LDL, insulin, and HOMA-IR and decreased HDL levels [30]. The first limitation is that we carried out only two evaluations of the patients, pre- and post-surgery 6 (4–8 years); therefore, we do not have data on the changes in BS outcomes over a short period of time (6 or 12 months, for example). Another limitation is the small sample size, which could not influence the lack of association of rs17782313 *MC4R* with anthropometric changes after BS, and the lack of an interaction effect of rs9939609 by bariatric surgery on weight and BMI.

## 5. Conclusions

In this cohort, after 6 (4–8) years of bariatric surgery, we showed that carriers of the rs9939609 and rs1421085 risk genotypes had greater post-surgery weight. Persons with the rs9930506 and rs1421085 risk genotypes lost less weight (TBWL). Insulin and insulin resistance levels were greater in patients with the three *FTO* polymorphisms. The carriers of the rs9939609 and rs1421085 risk haplotypes also lost less weight (TBWL) and less excess body weight (%EBWL) than did those of the other haplotypes. In carriers of the rs9939609 and rs9930506 risk haplotypes, TBWL decreased, and insulin and HOMA-IR levels were greater than those in carriers of the other haplotypes. These data suggest interaction effects of these polymorphisms of the *FTO* gene. Analysis additionally confirmed the interaction effects of the rs9930506 and rs1421085 risk genotypes on weight and BMI in response to bariatric surgery. These results could be used as a screening tool prior to bariatric surgery to help clinicians predict weight loss in patients with severe obesity and could be used prior to surgery to predict the success of bariatric surgery. Continuous work in the surgical management of obesity to understand the genetic and epigenetic factors and their role in obesity development and weight loss response is needed.

## Figures and Tables

**Table 1:** Anthropometric, clinical, and metabolic characteristics of patients at 6 years after bariatric surgery (n = 101).

Age (years)	47 (40–54)
Sex m/f	23/78
Weight (kg)	82 (73–103)
Height (m)	1.60 (1.53–1.66)
BMI (kg/m^2^)	33.8 (29.1–38.8)
SBP (mmHg)	122 (112–132)
DBP (mmHg)	74 (60–80)
TBWL (kg)	34.7 (22.9–48.9)
%TBWL	23 (16.6–33)
%EBWL	58.5 (40–79)
Glucose (mmol/L)	4.88 (4.6–5.2)
Total cholesterol (mmol/L)	3.95 (3.43–4.49)
HDL-cholesterol (mmol/L))	1.39 (1.16–1.6)
LDL-cholesterol (mmol/L)	2.0 (1.87–2.22)
Triglycerides (mmol/L)	1.06 (0.88–1.48)
Total protein (g/L)	7.0 (6.7–7.3)
Serum albumin (g/L)	3.9 (3.8–4.1)
AST U/L	27 (23–33)
ALT U/L	23 (18–31)
Alkaline phosphatase UI/L	87 (73–102)
Total bilirubin (µmm/L)	10.2 (8.5–11.9)
Indirect bilirubin (µmm/L)	6.8 (5.1–8.5)
Direct bilirubin (µmm/L)	3.4 (1.7–5.1)
Insulin µIU/L	12.6 (9.5–17.5))
HOMA-IR	2.73 (1.94–4.35)
Serum leptin (ng/mL)	25 (14.5–37.9)
Serum ghrelin (pg/mL)	322 (270–506)

The values are expressed as the median and interquartile range; SBP = systolic blood pressure; DBP = diastolic blood pressure; BMI = body mass index; TBWL = total body weight loss; %EBWL = percent of excess body weight loss; HDL = high-density proteins, HOMA-IR = homeostasis model assessment of insulin resistance; AST = aspartate aminotransferase; ALT = alanine aminotransferase; %TBWL = percent of total body weight loss.

**Table 2:** Distribution of polymorphism frequencies.

n = 101	Genotypic Frequency (%)	Allele	Allelic Frequency	X^2^	*p*
**rs9939609 *FTO* T>A**					
TT	40 (39.6)	T	0.63	0.011	NS
TA	47 (46.53)	A	0.37		
AA	14 (13.86)				
rs1421085 *FTO* T>C *					
TT	43 (42.57)	T	0.63	3.42	NS
TC	37 (36.63)	C	0.37		
CC	18 (17.82)				
**rs9930506 *FTO* A>G**					
AA	41 (41)	A	0.625	0.507	NS
AG	43 (43)	T	0.375		
GG	16 (16)				
**rs17782313 MC4R T>A**					
TT	78 (77.22)	T	0.87	0.214	NS
AT	21 (20.79)	A	0.13		
AA	2 (0.0198)				

A = adenine, C = cytosine; T = thymine; G = guanine; NS = not significant; * n = 98 participants.

**Table 3:** *FTO* polymorphism dominant model.

**rs9939609 T>A**				
	**TT n = 40**	**TA and AA n = 61**	**T**	***p***
Post-Surgery Weight (Kg)	84 ± 16.6	92.8 ± 24.2	−1.98	0.049
Serum Insulin µIU/L	12.0 ± 4.8	16.2 ± 9.2	−2.66	0.008
Serum Leptin (ng/mL)	24.5 ±16	32.5 ± 21	−2.01	0.049
HOMA-IR	2.69 ± 1.2	3.8 ± 2.6	−2.49	0.014
**rs9930506 A>G**				
	**AA n = 41**	**AG and GG n = 59**	**Z**	***p***
Pre-Surgery BMI (kg/m^2^)	50.3 (45.7–55.2)	45.3 (42.5–54.6)	1.91	0.05
TBWL (kg)	38.3 (25.2–59)	33 (18.6–41.8)	2.35	0.018
Total Cholesterol (mmol/L)	3.77 (3.2–4.34)	4.18 (3.6–4.7)	−2.09	0.036
Serum Insulin (µIU/L)	10.5 (7.5–13.4)	13.7 (10.5–9.4)	−3.53	0.0004
HOMA-IR	2.28 (1.7–2.92)	3 (2.18–4.9)	−3.47	0.0005
**rs1421085 T>C**				
	**TT n = 44**	**TC and CC n = 55**	**T**	***p***
Post-Surgery Weight (kg)	84 ± 16	93.9 ± 25	−2.29	0.023
TBWL (kg)	42.5 ± 20	32.5 ± 20	2.49	0.014
Serum Insulin (µIU/L)	12 ± 4.9	16.2 ± 9.3	−2.67	0.008
HOMA-IR	2.68 ± 1.3	3.82 ± 2.6	−2.58	0.011

BMI = body mass index; TBWL = total body weight loss; HOMA-IR = homeostasis model assessment insulin resistance; Z value from Mann–Whitney test; T value from Student’s *t* test.

**Table 4:** Analysis of the rs9939609 (T>A) and rs9930506 (A>G) haplotypes.

	Haplotype 1 TA (n = 32)	Haplotype 2 TG (n = 8)	Haplotype 3 AA (n = 9)	Haplotype 4 AG (n = 51)	F	*p*
TBWL (kg)	40.5 ± 20.8	39.4 ± 11	54.4 ± 20.8	31.5 ± 19	4.10	0.008
%EBWL	52.3 ± 20.9	66.6 ± 18	85.5 ± 29	56.8 ± 32	3.62	0.015
Insulin µIU/L	11.4 ± 4.7	12.6 ± 2.5	10.5 ± 4.6	17.2 ± 9	4.79	0.003
HOMA-IR	2.6 ± 1.2	2.74 ± 0.7	2.31 ± 1.3	4 ± 2.7	4.0	0.009

Total n = 100 participants. TBWL = total body weight loss; %EBWL = percent of excess body weight loss; HOMA-IR = homeostasis model assessment insulin resistance; haplotype 4 ≠ 1 and 3; haplotype 3 ≠ 1 and 4 haplotypes; serum insulin and HOMA-IR of haplotype 4 ≠ 1 and 3. ANOVA test.

**Table 5:** The *FTO* genotypes and response to bariatric surgery (variance analysis of repeated measures of one factor).

Interaction of Genotype on Weight and BMI					
**rs9939609 T>A**					
	**TT n = 40**		**TA and AA n = 61**		***p***
	**Before Surgery**	**After Surgery**	**Before Surgery**	**After Surgery**	**Genotype**
Weight (kg)	124 ± 23	84 ± 16.6 *	128 ± 28	92.8 ± 24.2 *	0.205
BMI (kg/m^2^)	49.6 ± 9	33.0 ± 5.6 *	49 ± 9.5	35.6 ± 8 *	0.118
**rs9930506 A>G**					
	**AA n = 41**		**AG and GG n = 59**		***p***
	**Before Surgery**	**After Surgery**	**Before Surgery**	**After Surgery**	**Genotype**
Weight (kg)	128.5 ± 25.5 121 (117–140)	85 ± 16 * 81.7 (76–93.9)	125.4 ± 26.3 121 (106–140)	92.8 ± 24.6 * 89 (73–110)	0.007
BMI (kg/m^2^)	51.7 ± 10.8 50.3 (45.7–55.2)	34 ± 6.2 * 33.8 (29–38.5)	47.9 ± 8 45.3 (42.5–54.6)	35.3 ± 7.8 * 34 (29.3–39.4)	0.002
**rs1421085 T>C**					
	**TT n = 44**		**TC and CC n = 55**		***p***
	**Before Surgery**	**After Surgery**	**Before Surgery**	**After Surgery**	**Genotype**
Weight (kg)	126.4 ± 23.7 12 1 (116–138)	84 ± 16 * 80.4 (73–94.5)	126.4 ± 28 121 (106–142)	93.9 ± 25 * 91.7 (73.3–111)	0.014
BMI (kg/m^2^)	50.6 ± 10 50 (44.5–54.3)	33.3 ± 6 * 33.2 (29–38.2)	48.4 ± 9 46 (41.3–54.8)	35.8 ± 8 * 35.5 (29.6–40.7)	0.005

The data are presented as the median ± ED and median 25–75 quartile. BMI = body mass index. *p* value = genotype interaction and variance analysis of repeated measures of one factor. * *p* < 0.00001 intragroup difference before and after bariatric surgery.

## Data Availability

Data analyzed during this study are included in this article. Further inquiries can be directed to the corresponding author.
